# Incremental net benefit of wearable devices for home monitoring of chronically ill patients

**DOI:** 10.1093/eurpub/ckac129.012

**Published:** 2022-10-25

**Authors:** G Altamura, MC Nurchis, G Santoli, MT Riccardi, M Sapienza, G Sessa, G Damiani

**Affiliations:** 1 Department of Life Sciences and Public Health, Università Cattolica del Sacro Cuore, Rome, Italy; 2 Department of Woman, Child Health and Public Health, Fondazione Policlinico Universitario A. Gemelli, Rome, Italy

## Abstract

**Background:**

Hospital overcrowding is a growing problem worldwide. Studies demonstrated that up to 40% to 67% of hospitalizations of residents in nursing homes may be avoidable, causing health and economic damages. Furthermore, research shows that for non-critical patients there are arguably no differences between home and hospital recovery in terms of health outcomes, with a preference for home settings in most patients. During COVID-19 pandemic, telemedicine and homecare increased its range of possible intervention, allowing efficient and cost-effective processes of care. Transdermal sensors are indeed a cheap and easy to use alternative to conventional instruments, allowing a continuously operative and ready-to-use tool to care providers. This systematic review aims to map the application fields of these technologies, demonstrating their accuracy and assessing their cost-effectiveness in chronically ill home-assisted patients.

**Methods:**

Articles were retrieved from Scopus, Web of Science, and PubMed. The dominance ranking matrix (DRM) tool was applied to allow a qualitative synthesis of the studies. Incremental net benefits (INBs) were estimated and meta-analysis was implemented to pool INBs across studies. A comparison between wearables and conventional tools accuracy was simultaneously carried out through a literature review.

**Results:**

The database search identified 1156 publications of which six articles were considered eligible for the meta-analysis. According to DRM, 80% of evaluated studies showed the cost-effectiveness of wearable devices. The pooled INB of wearables over conventional measurement was estimated at US$1280 (95% CI US$952 - US$2849). In 85% of evaluated wearables the accuracy resulted comparable to conventional measurement tools.

**Conclusions:**

Wearables performances resulted as accurate as conventional methods and their application cost-effective. A continuous measurement of parameters may relate to a better process of care for chronically ill outpatients.

**Key messages:**

• Wearables are a cheap and accurate alternative to conventional life parameters measurement tools.

• Technology evolution might soon reduce the pressure on hospitals, changing the care process of chronically ill outpatients allowing continuous evaluation of their health status.

